# Editorial: Artificial intelligence and machine learning in pediatrics

**DOI:** 10.3389/frai.2026.1814141

**Published:** 2026-03-18

**Authors:** Kaya Kuru, Noreen Caswell, Gregory R. Hart, Jennifer Quon, Louis Ehwerhemuepha

**Affiliations:** 1 School of Engineering & Computer Science, University of Central Lancashire, Preston, United Kingdom; 2 Software Engineering Department, Fenerbahce Universitesi, Ataşehir, Türkiye; 3 University of Guam, Mangilao, GU, United States; 4 The Hospital for Sick Children Paediatric Emergency Medicine, Toronto, ON, Canada; 5 Children's Hospital of Orange County, Orange, CA, United States

**Keywords:** artificial intelligence, big data analytics, large language models, machine learning, natural language processing, pediatrics

## 1 Introduction

Pediatrics is a branch of medicine that specializes in the medical care of infants, children, and adolescents, who undergo rapid developmental changes and exhibit distinct disease patterns. The development of modern technologies like biosensors (Kuru et al., 2020), wearable sensors (Caswell et al., 2020), medical imaging, well-designed laboratory facilities, and genetics advancements has led to a huge amount of data with multidimensional and complex structures in different formats in pediatrics, which exceeds traditional human analytical capabilities. A massive amount of childcare data is generated every second (Kuru, 2021). These vast volumes of pediatric data indicate the need to develop intelligent technologies to efficiently exploit this wealth of resources. Artificial Intelligence (AI) and Machine Learning (ML) technologies can be used to efficiently process large and complex datasets composed of a diverse set of medical data, such as electronic health records, images, genetic data, and wearable sensor data, to make precise, individualized, and data-driven pediatric care possible.

## 2 Artificial intelligence and machine learning in pediatrics

Recent research shows that AI/ML can discover new approaches to representing multidimensional, nonlinear relationships and patterns in children's data. More specifically, it can help with providing a rather quick and accurate understanding of effective treatment strategies and further improving clinical decision-making. Models based on AI technology show impressive efficacy in early diagnostics of pediatric conditions like congenital heart disease, childhood cancers, and rare genetic disorders. Among the recent trends in medical imaging (e.g., radiology, pathology, and ophthalmology), deep learning (DL) techniques are used to allow for faster and more accurate identification of anomalies, which often reach or surpass those performed by specialists. However, even the diagnostics have provided a very clear picture of how crucial it is to carry out additional research in employing AI/ML algorithms. There is still a large gap and numerous other obstacles to integrating AI/ML into pediatric health care. The main challenges and guardrails can be summarized as follows

- Age stratification and developmental change (non-stationarity across growth stages).
- Smaller datasets/rarer outcomes and resulting overfitting risk.
- Equity and bias (race/ethnicity, language, disability, access).
- Lack of standardized pediatric data ontologies.
- Consent/privacy complexities for minors and family context.
- Prospective evaluation and deployment (monitoring drift, safety, human factors).

Driven by the exponential growth of complex pediatric data, this Research Topic explores the potential role of AI/ML in pediatrics, particularly concentrating on harnessing big data analytics to enhance children's health outcomes. Finding out how AI/ML may improve the early diagnosis, treatment, and prognosis of pediatric disorders, as well as testing theories about the effectiveness of AI/ML-driven precision medicine in the provision of pediatric care, are among the main objectives. Can AI help develop novel drugs or approaches to cure pediatric diseases? Through asking these questions, the topic attempts to obtain (i) a complete picture of what is possible and (ii) the limitations of AI/ML in pediatric healthcare, ultimately leading the way toward safer, more effective and personalized medical treatment for children.

## 3 Innovative AI studies in pediatric practices

Against the above backdrop, this Research Topic of the journal was convened by both the pediatricians and AI specialists to create a forum which presents conducted scholarly studies focused on recently arising issues in pediatric healthcare. In all, 14 top-notch articles have been included in this Research Topic through rigorous review and selection. Carvalho et al. carry out an investigation on ML techniques regarding their performance in predicting suicide related behaviors among young populations within a lower-middle-income country context- particularly with respect to pediatric psychiatric emergencies in Brazil. The research demonstrates strong potential for identifying suicidal risk in pediatric emergency settings, especially useful in regions with limited specialized psychiatric resources, by integrating clinical data with social determinants. On a separate note, traditional drug dosing approaches are often insufficient to ensure effective therapy, exposing patients to the risk of overdosing, leading to adverse effects with potential therapeutic failure (Anderson and Holford, 2008). To find a practical solution to this common problem, Frasca et al. examine the application of ML to optimize antibiotic dosing in pediatric patients, focusing on predicting the ceftaroline dose. Findings in their research suggest that carefully curated AI/ML models can outperform conventional ceftaroline dosing strategies in pediatric patients. Based on the most recent developments in Large Language models (LLM), Vertesich et al. investigated the quality, accuracy and reliability of inference that can be made from a LLM (ChatGPT) for parental education in a pediatric medical context of developmental dysplasia of the hip (DDH) specifically. The study concludes that LLMs can present high-quality answers to questions about DDH and may serve as a useful supplementary information resource for parents. In another direction, Wang et al. are looking at how much air pollution exposure and surgical site infection (SSIs) are related among adolescents undergoing orthopedic surgery, using Shanghai as a case study. The study demonstrates that DL models can readily identify the dominant predictors, supporting perioperative air-quality interventions. Likewise, genetic disorders are affecting children and their families drastically (Kuru et al., 2014). Zhao et al. present findings about the role and potential of AI, particularly ML/DL, in enhancing the objective measurement of attention deficit hyperactivity disorder (ADHD) treatment outcomes. More specifically, the significant potential of AI in tasks such as early screening and risk prediction, diagnostic assistance and classification, precise differential diagnosis, symptom severity quantification, and the identification of heterogeneous ADHD subtypes was highlighted. Similarly, Tang et al., in their work, describe how to find diagnostic markers and molecular subtypes for pediatric Crohn's disease (PCD) from gene expression data using ML techniques. Around 1.7 billion diarrhea cases in children are reported every year (WHO, 2024). It is the third leading cause of death in children under 5 years old and is responsible for killing around 443,832 children every year (WHO, 2024). Yehuala et al. focus on the factors that determine the child feeding practices during episodes of diarrhea in children in East Africa based on a huge population survey data with the help of ML models. Considering the integration of intelligent devices into child healthcare practices, Fan et al. examine whether home spirometry telemonitoring for children with asthma is possible, trustworthy, and acceptable, and assess real-world issues affecting its clinical integration. Similarly, Soualmi et al. present the development and implementation of an automated instrument (AGMA-PESS) for the purpose of improving the efficiency of General Movement Assessment (GMA) in preterm infants. On the other hand, nocturnal enuresis (NE) is a widespread disease among children, and a proper cure has yet to be found to treat it (Kuru et al., 2024, 2018). Liao et al. discuss how health education improves the quality of life among children receiving treatment for NE and their caregivers using data mining techniques. In the same way, Ye et al. describe an application of ML for urinary tract infection (UTI) risk prediction in febrile children under 3 years of age. In the article of Zhang et al., a review is made concerning the current status and clinical applicability of AI/ML for bronchopulmonary dysplasia (BPD) predictive measures in pre-term infants. Tozzi et al. discuss the position of AI in the contemporary focus on pediatric brain tumors at a European level with respect to data sources and generalisability of AI algorithms. When implementing AI-based technologies in childcare, it is highly crucial to incorporate the perspectives of patients and their families into all phases (Haan et al., 2019) to make them successful (Caswell et al., 2020), concerning their acceptability. To guide the AI developer in childcare, Huang et al. investigate the perception, knowledge, and attitude of parents regarding AI applications in pediatric practice.

## 4 Conclusion

The continuous evolution of AI is equipping pediatricians with new tools to perform their tasks more efficiently. In this direction, this topic delves into the advancement of AI in pediatric medicine. Based on the findings in this Research Topic, it can be safely concluded that AI/ML applications are increasingly transforming pediatric healthcare by enabling data-driven decision-making, enhancing diagnostic accuracy, personalizing treatments, proactive interventions and streamlining clinical workflows. Further developments in AI will significantly shape the future of pediatrics in the years ahead, paving the way for better healthcare for pediatric patients and their families through informed clinical decision-making. While significant challenges remain, ongoing research, ethical oversight, and interdisciplinary collaboration are essential to realize and exploit the full potential of AI-driven innovations, ultimately improving the health and wellbeing of children worldwide.

While AI is providing invaluable guidance to pediatricians and iincreasingly playing a pivotal role in childcare starting from the prenatal phase, future research should focus on developing explainable and trustworthy AI models, creating large-scale, reliable pediatric-specific datasets and establishing robust validation and regulatory frameworks. Collaborative efforts among clinicians, data scientists, policymakers, and ethicists will be essential to ensure responsible and effective adoption of AI in pediatric healthcare.
